# Brain Volumes and Developmental Outcome in Childhood Following Fetal Growth Restriction Leading to Very Preterm Birth

**DOI:** 10.3389/fphys.2018.01583

**Published:** 2018-11-16

**Authors:** Eva Morsing, Mariya Malova, Anna Kahn, Jimmy Lätt, Isabella M. Björkman-Burtscher, Karel Maršál, David Ley

**Affiliations:** ^1^Department of Pediatrics, Clinical Sciences Lund, Lund University, Lund, Sweden; ^2^Neonatal Intensive Care Unit, Istituto Giannina Gaslini, Genoa, Italy; ^3^Department of Radiology, Clinical Sciences Lund, Lund University, Lund, Sweden; ^4^Department of Medical Imaging and Physiology, Skåne University Hospital, Lund, Sweden; ^5^Department of Obstetrics and Gynecology, Clinical Sciences Lund, Lund University, Lund, Sweden

**Keywords:** brain volumes, magnetic resonance imaging, neuro-development, preterm birth, fetal growth restriction

## Abstract

**Background:** Children born very preterm (PT) after fetal growth restriction (FGR) exhibit cognitive impairment at early school age. The relationship between neurodevelopmental impairment and attained regional brain volumes is unknown.

**Methods:** We studied 23 preterm children with FGR (PT-FGR), 24 matched preterm children AGA (PT-AGA), and 27 matched term AGA children (T-AGA) by measuring brain volumes with magnetic resonance imaging at early school age. Cognitive and motor functions were assessed by the Wechsler Intelligence Scales for Children and the ABC-Movement score.

**Results:** The mean (*SD*) full-scale IQ was 80 (17) in the PT-FGR group and 103 (12) in the PT-AGA group (*p* < 0.001). The PT-FGR group had lower mean total, gray matter, white matter, thalamic, cerebellar white matter, and hippocampal volumes as compared to the T-AGA group (*p* = 0.01, 0.04, 0.003, 0.002, 0.001, and 0.009, respectively). Brain volumes did not differ significantly between the PT groups. Reduction of hippocampal volume correlated with degree of growth restriction at birth (*r* = 0.46, *p* = 0.05). Neither the full-scale IQ nor the ABC movement score <5th percentile were related to brain volumes.

**Conclusion:** Brain volumes as determined by MRI at early school age were primarily associated with degree of prematurity at birth and less with FGR. Regional brain volumes did not discriminate cognitive and motor function beyond that predicted by gestational age at birth.

## Introduction

Fetal growth restriction (FGR) at early GA is associated with high risk of perinatal mortality, morbidity, and long-term neurodevelopmental impairment (Torrance et al., 2010; Baschat, 2011). ARED blood flow in the umbilical artery assessed by Doppler velocimetry is associated with adverse outcome in preterm FGR (PT-FGR) fetuses (Hartung et al., 2005). No consensus on clinical management of FGR fetuses diagnosed in the second trimester (early-onset) has been achieved so far (Baschat and Odibo, 2011). Prenatal clinical management has to consider the high risk of neonatal complications associated with severe prematurity against the substantial risk of fetal death.

Previous studies, with the objective of relating impaired fetal growth to different aspects of outcome have mainly used birthweight small for GA, (SGA) as a proxy and a common denominator for FGR. As well known, this definition is imprecise and will include constitutionally small infants as well as truly growth-retarded infants. The objective of the present study is to evaluate long-term effects of FGR leading to very preterm birth (<30 GW). The patho-physiological consequences of early-onset FGR, i.e., leading to very preterm birth, are sensitively detected by fetal blood flow evaluation using Doppler ultrasound (Figueras and Gratacos, 2014).

Infants born very preterm (PT) were reported to exhibit reduction in total and regional cerebral volumes determined by MRI (Keunen et al., 2012) and the observed brain volume reduction has been shown to persist into childhood and adolescence (Reiss et al., 2004; Northam et al., 2011). In fetuses affected by late-onset growth restriction, fetal MRI showed different cortical development as compared to fetuses with normal growth (Egana-Ugrinovic et al., 2013). Subjects with late-onset FGR, as determined by birthweight small for GA and born at term age, exhibited a global reduction in brain volume as well as regional reductions in cortical surface area (Ostgard et al., 2014).

Fetal growth restriction leading to very preterm birth, i.e., early-onset FGR, has been associated with altered brain structure and lower brain volumes during the PT period and at term-equivalent age (Tolsa et al., 2004; Thompson et al., 2007). Interpretation of previous studies on FGR and brain volumes is complicated by the heterogeneity of study designs and inclusion criteria. Some authors have described reduced brain volumes in small for GA subjects born at late gestation with no information on preceding fetal blood flow examinations (Ostgard et al., 2014). Differences in age of subjects at MRI assessment also complicate comparisons of brain volumes studies, since brain growth changes with age in healthy subjects (Sussman et al., 2016).

To date, there are no controlled studies investigating the effect of FGR leading to very preterm birth on MRI-assessed regional brain volumes in children; thus, it is unclear whether the alterations in brain volumes persist into childhood. Infants with FGR and ARED flow leading to very preterm birth have an increased neonatal morbidity and an adverse cognitive outcome at school age (Brodszki et al., 2009; Morsing et al., 2011). Clinical guide-lines for management of this group of fetuses still differ between centers and countries. It is therefore essential, from a neuro-scientific as from a clinical point of view, to investigate brain development in this well-defined group of subjects. The aim of this study was to evaluate regional brain volumes and their relationship to cognitive and motor outcome at 8 years of age in children born very preterm after FGR and ARED umbilical artery blood flow.

## Patients and Methods

### Population

The present work is part of a prospective case-control follow-up study of children born very preterm (<30 gestational weeks) after early-onset FGR and ARED blood flow in the umbilical artery. Doppler flow velocity signals from the umbilical artery, the middle cerebral artery, DV, umbilical veins and from both maternal uterine arteries were recorded using Philips HDI 5000 or Philips IU22 (Philips Medical Systems, Bothell, WA, United States) ultrasound systems. Abnormal DV flow was defined as absent or reverse flow during the a-wave. The Doppler velocimetry was in all cases performed in a standardized way by experienced sonographers. FHR recordings and Doppler velocimetry were performed daily. Between 1998 and 2004, 42 such live-born neonates were delivered on fetal indication at the level III perinatal center at Skane University Hospital in Lund, Sweden. Since 1998, a proactive clinical management protocol is used for management of very preterm fetuses with FGR aiming to avoid severe fetal hypoxia and deterioration of fetal condition. The protocol indicates delivery at the occurrence of reversed end-diastolic (RED) flow in the umbilical artery, and/or if there are rapidly progressing changes in the ductus venosus (DV) blood velocity waveform or pathological changes of fetal heart rate (FHR; loss of variability, late decelerations). After 26 gestational weeks, absent end-diastolic (AED) flow in the umbilical artery was indication for delivery after the full course of antenatal steroid treatment. The clinical protocol included delivery by cesarean section and neonatal active care comprising early surfactant treatment and early enteral feeding with human milk. During the same period, two control groups with birth weight AGA were identified; one group consisted of preterm infants matched for GA and sex (PT-AGA), and the other group was born at term matched for sex and age at examination (T-AGA). At the age of 7–8 years, follow-up examinations were performed. Perinatal data, prevalence of cerebral palsy, as well as neurocognitive, cardiovascular and pulmonary outcome in this cohort have been reported previously (Brodszki et al., 2009; Morsing et al., 2011, 2012, 2014).

Children from the three groups that underwent an examination with brain MRI at 8 years of age constituted the index group (PT-FGR, *n* = 23) and two control groups (PT-AGA, *n* = 24; T-AGA, *n* = 27) of the present study.

### Movement ABC

Children were evaluated with The Movement Assessments Battery for Children 2nd edition (Movement ABC) consisting of eight items divided into three subtests: manual dexterity, ball skills, and static/dynamic balance. (Henderson and Barnett, 2007) High scores denote motor performance deficits. Scores below the 5th percentile indicate severe motor impairment, and scores between the 5th and 15th percentile indicate minor motor impairment.

### Cognitive Tests and Behavior Questionnaire

Cognitive evaluation was performed by Wechsler scales (the Wechsler Preschool and Primary Scale of Intelligence-III and the Wechsler Intelligence Scale for Children-III, 1991 revision, British version) at 5–8 years of age (range 60–105 months). (Wechsler, 1991, 2002; Brown, 2001) Both tests consist of two IQ subscales, VIQ and PIQ, forming the full-scale IQ (FIQ). All scales have a mean of 100 points and *SD* of 15. Cognitive impairment was defined as FIQ < 70 (>2 *SD* below the normative mean).

During follow-up visit, the parents were interviewed and filled in two scoring questionnaires regarding attention-deficit disorder (ADD; Brown’s ADD scales)(Brown, 2001) and behavior problems (Strengths and Difficulties Questionnaire (SDQ). Brown’s ADD consists of 44 items and includes tasks examining ability to sustain attention and energy, effort to complete tasks, to regulate moods and recall learned material. A score >55 indicates risk for ADD. The SDQ is a behavioral screening questionnaire that comprises 25 items divided into subscales (prosocial, hyperactivity, emotional problems, conduct, and peer problems). A total score of 13 was considered to be borderline or high.

### MRI Scanning

All children were informed about the MRI procedure beforehand and were awake during the scanning. None of the children were sedated. Ear plugs and headphones were used for hearing protection and children could watch a movie during the examination by means of projection on a screen behind the scanner and viewed through a mirror mounted to the head coil.

The MRI investigations were performed on a 3T scanner (Achieva, Philips, Best, Netherlands) with an eight channel SENSE head coil. Volumetric data were acquired using a T1 3D magnetization prepared rapid acquisition with gradient echo (MPRAGE) sequence with isotropic voxel size of 1 mm^3^, allowing reconstruction in any plane, repetition time = 9 ms, echo time = 4 ms, and flip angle = 10°. Reconstruction and segmentation of the brain was performed with the FreeSurfer image analysis suite^1^. Volumetric data were acquired for total intracranial volume, GM, WM, cerebrospinal fluid, cerebellar GM and WM, and thalamus. All segmentations were subsequently reviewed and, if necessary, the automatically segmented volumes were adjusted manually using in-house MatLab (Mathworks, MA, United States) based software. In particular, the anterior border of the cerebellar WM was adjusted to consequently exclude brainstem areas. The peduncles of the cerebellum were included in the WM volumes and the vermis was included in the GM volumes.

Hippocampal segmentation was performed manually on a PACS workstation (IDS7, Sectra, Linköping, Sweden) in consensus by two radiologists blinded to perinatal data. T1 MPRAGE images were separately for both sides reformatted to an oblique coronal viewing plane perpendicular to the long axis and in parallel to the anterior and posterior limits of the hippocampus. Delineation was performed in a posterior to anterior direction with the plane showing the crus of the fornix defining the level of the most posterior aspect of the hippocampus and the plane where the temporal horn appears medially and beneath the amygdala the most anterior portion. As further boundaries of the hippocampus we used the alveus (superior), WM of the para-hippocampal gyrus (inferior), the temporal horn of the lateral ventricle (lateral), and the ambient cistern (medial). The subiculum was included in the hippocampus. Anteriorly the temporal horn of the lateral ventricle and the alveus were used to separate hippocampus from amygdala. When in doubt, an imaginary line between the ambient cistern and the middle of the temporal horn of the lateral ventricle was used. ROIs were first drawn by observer 1 according to the protocol, then observer 2, in a separate session defined visually the boundaries of the hippocampi on unmarked projections ant then checked ROIs for mismatches. All suggested changes of hippocampal delineation were discussed in consensus between the observers and then implemented.

### Data Collection and Analysis

Demographic data and clinical parameters, including prenatal, perinatal, and neonatal data were collected from the obstetric and pediatric patient records. Head circumference was measured by a pediatrician at the follow-up examination. Information on socioeconomic factors was obtained from questionnaires administered to the parents while the children attended the tests. Cognitive, motor, and behavioral test results, as well as MRI data were registered. Possible associations between the volume of brain structures, clinical data and neurobehavioral test results were assessed.

Statistical analyses were performed using SPSS 23.0 statistical software (SPSS Inc, Chicago, IL, United States). Categorical variables were compared between groups by the χ^2^ test. Differences in continuous variables were assessed with two-way analysis of variance (ANOVA) with *post hoc* Bonferroni correction for multiple group comparisons. *P*-values of <0.05 were considered statistically significant. Confounders were explored by linear regression analysis.

## Results

### Perinatal Clinical Data and Postnatal Morbidity

Birth characteristics, neonatal morbidity, and rate of cerebral palsy in the three groups are presented in Table 1. All 23 infants of the index group had ultrasonically estimated fetal weight more than 2 *SD* below the mean of the Swedish reference population (Marsal et al., 1996). Eighteen fetuses had absent, and five had reversed end-diastolic blood flow in the umbilical artery. Fifteen fetuses out of 23 had signs of brain-sparing, i.e., middle cerebral artery pulsatility index >2 *SD* below the GA related mean (Mari and Deter, 1992). The mean (*SD*) GA at birth was 26.4 (1.5) weeks and all infants were small-for-GA with birthweight < mean−2 *SD* of the reference (Marsal et al., 1996).

**Table 1:** Birth characteristics, neonatal morbidity, and rate of cerebral palsy.

				Significance of difference		
	PT-FGR (*n* = 23)	PT-AGA (*n* = 24)	T-AGA (*n* = 27)	PT-FGR–PT-AGA	PT-FGR– T-AGA	PT-AGA – T-AGA
Gestational age weeks, mean ± SD	26.4 ± 1.5	26.7 ± 1.6	39.4 ± 0.7	*ns*	*<0.001*	*<0.001*
ARED flow in the umbilical artery, *n* (%)	23 (100)	0	0	*<0.001*	*<0.001*	*na*
Birthweight g, median (range)	664 (395–976)	1032 (660–1790)	3550 (3000–4390)	*<0.001*	*<0.001*	*<0.001*
Birthweight deviation %, median (range)	−37 (−63 – −23)	−5 (−22 –+14)	−1 (−17 –+29)	*<0.001*	*<0.001*	*0.05*
Apgar score < 7 at 5 min *n* (%)	1 (4)	4 (17)	0	*ns*		
Cesarean section *n* (%)	23 (100)	14 (58)	0	*<0.001*		
Sex, female/male *n*	13/10	11/13	14/13	*ns*	*ns*	*ns*
Term head circumference cm, mean ± SD	34.4 ± 1.5	35.6 ± 1.0	35.5 ± 1.2	*0.02*	*0.02*	*ns*
IVH III/PVHI *n* (%)	1 (4)	2 (8)	0	*ns*	
ROP *n* (%)	4 (17)	6 (25)	0	*ns*	
NEC *n* (%)	3 (13)	0	0	*ns*	
BPD *n* (%)	16 (70)	6 (25)	0	*0.003*	
Postnatal steroid treatment *n* (%)	9 (39)	5 (21)	0	*ns*	
Septicemia *n* (%)	14 (61)	6 (25)	0	0.02	
Cerebral palsy *n* (%)	2 (9)	4 (17)	0	*ns*	

*PT, preterm; T, term; AGA, appropriate for gestational age; birthweight deviation % of expected birthweight according to Swedish standard; FGR, fetal growth restriction; ARED, absent or reversed end-diastolic blood flow; ns, non-significant; IVH, Intraventricular hemorrhage; PVHI, peri-ventricular hemorrhagic infarction; ROP, retinopathy of prematurity; NEC, necrotizing enterocolitis; BPD, bronchopulmonary dysplasia.*

Infants in the control groups (PT-AGA and T-AGA) had AGA birth weight. Antenatal Doppler velocimetry was not performed in the control children.

The Apgar score <7 at 5 min and sex distribution were similar in the preterm groups. Prevalence of severe intraventricular hemorrhage, ROP and necrotizing enterocolitis, as well as use of postnatal steroids was similar in the preterm groups. The PT-FGR group had higher rate of septicemia and BPD than the control PT-AGA group.

### Cognitive Function, Motor Performance, and Behavior

Results of cognitive evaluation, motor performance, ADD, and behavior are shown in Table 2. The PT-FGR group had lower motor performance score, higher scores in attention deficit and behavior questionnaires compared to the T-AGA group. Scores from Brown’s ADD and SDQ scales did not differ between the two preterm groups. PIQ and FIQ were lower in both preterm groups compared to the T-AGA group. FIQ was lower in the PT-FGR group than in the PT-AGA group.

**Table 2:** Cognitive evaluation by WISC-III/WPPSI-III, motor performance by ABC-movement, attention deficit disorder and behavior by Brown’s ADD and SDQ and measurements of head circumference at 5–8 years.

				Significance of difference		
	PT-FGR (*n* = 23)	PT-AGA (*n* = 24)	T-AGA (*n* = 27)	PT-FGR – PT-AGA	PT-FGR – T-AGA	PT-AGA – T-AGA
**Parental education**						
High school/university						
Mother, *n* (%)	9/23 (39)	12/23 (52)	14/26 (54)	*ns*	*ns*	*ns*
Father, *n* (%)	5/23 (22)	8/23 (35)	11/26 (42)	*ns*	*ns*	*ns*
**Verbal IQ**	85 ± 18	95 ± 14	102 ± 11	*ns*	*<0.001*	*ns*
mean ± SD						
**Performance IQ**	80 ± 16	87 ± 15	105 ± 15	*ns*	*<0.001*	*<0.001*
mean ± SD						
**Full scale IQ**	80 ± 17	90 ± 13	103 ± 12	*0.05*	*<0.001*	*0.003*
mean ± SD						
**Motor performance**	8 (34)	3 (12)	1 (4)	*ns*	*0.002*	*ns*
**< 5th percentile**, *n* (%)						
**ADD combined**	51.4 ± 10.9	48.1 ± 10.1	42.1 ± 5.6	*ns*	*0.002*	*ns*
mean ± SD						
**ADD combined > 55**, *n* (%)	8 (35)	7 (29)	1 (4)	*ns*	*0.007*	*0.019*
**SDQ total**, mean ± SD	9.6 ± 5.1	7.2 ± 5.9	4.4 ± 4.9	*ns*	*<0.001*	*ns*
**SDQ total > 13**, *n* (%)	6 (26)	4 (17)	1 (4)	*ns*	*0.04*	*ns*
**Head circumference**	51.7 ± 1.6	52.9 ± 1.6	54.6 ± 1.6	*ns*	*<0.001*	*0.009*
**at MRI**, cm, mean ± SD						

*WISC, Wechsler Intelligence Scale for Children; 3rd edition. WPPSI, Wechsler Preschool and Primary Scale of Intelligence; 3rd edition. ABC-movement, Movement assessments battery for children; Brown’s ADD, attention deficit disorder; SDQ, strength and difficulties questionnaire; PT, preterm; AGA, appropriate for gestational age; FGR, fetal growth restriction; MRI, magnetic resonance imaging.*

#### Subjects Not Examined With MRI

The numbers of subjects evaluated for cognitive and behavioral outcome at 6–8 years of age in the background population (Morsing et al., 2011) but not examined by MRI were 11, 10, and 7 in the background PT-FGR, PT-AGA, and the T-AGA groups, respectively. Birthweight deviation and GA at birth, as well as the cognitive outcome, behavior, and motor performance did not differ between the infants examined and those not examined with MRI.

### MRI Brain Volumes

**FIGURE 1:**
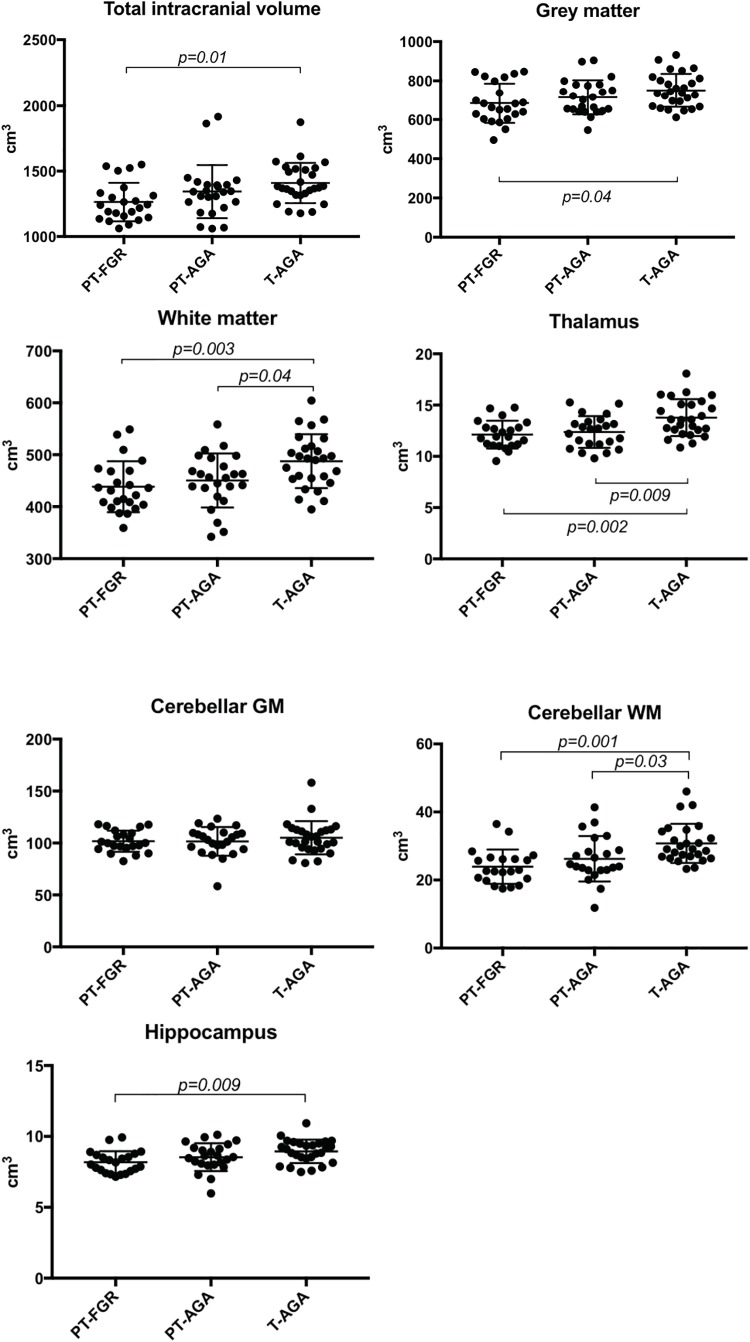
Dot plots describing individual values of total intracranial brain volume and regional brain volumes in subjects with fetal growth restriction and very preterm birth (PT-FGR group), very preterm birth and birthweight appropriate for gestational age (PT-AGA group), and birthweight appropriate for GA at term age (T-AGA group). Horizontal lines depict group means and standard deviations. Cerebellar GM, cerebellar gray matter; Cerebellar WM, cerebellar white matter.

The PT-FGR group had significantly lower mean total intracranial, GM and WM, thalamic, cerebellar WM and hippocampal volumes as compared to the T-AGA group (Table 3 and Figure 1). The PT-AGA group had lower mean WM, thalamic and cerebellar WM volumes as compared to the T-AGA group. Brain volumes did not differ significantly between the PT-FGR and the PT-AGA groups (Table 3 and Figure 1). When compared to term controls, female FGR subjects had smaller thalamic, cerebellar WM and hippocampal volumes, while males had smaller WM volumes.

**Table 3:** Brain volumes (cm^3^, mean ± SD).

Brain structure		Group		Significance of difference		

cm^3^	PT-FGR (*n* = 23)	PT-AGA (*n* = 24)	T-AGA (*n* = 27)	PT-FGR–PT-AGA	PT-FGR – T-AGA	PT-AGA – T-AGA
**Total intracranial volume**	1264 ± 147	1344 ± 203	1409 ± 153	*ns*	*0.01*	*ns*
male	1287 ± 153	1434 ± 213	1462 ± 179	*ns*	*ns*	*ns*
female	1246 ± 146	1237 ± 130	1361 ± 111	*ns*	*ns*	*ns*
**Gray matter**	684 ± 101	714 ± 88	750 ± 85	*ns*	*0.04*	*ns*
male	705 ± 119	747 ± 88	777 ± 102	*ns*	*ns*	*ns*
female	669 ± 87	676 ± 75	726 ± 59	*ns*	*ns*	*ns*
**White matter**	438 ± 49	450 ± 52	488 ± 52	*ns*	*0.003*	*0.04*
male	449 ± 52	471 ± 39	509 ± 50	*ns*	*0.02*	*ns*
female	431 ± 46	426 ± 55	468 ± 46	*ns*	*ns*	*ns*
**Thalamus**	12.1 ± 1.4	12.4 ± 1.5	13.8 ± 1.8	*ns*	*0.002*	*0.009*
male	12.4 ± 1.1	12.7 ± 1.8	14.1 ± 2.3	*ns*	*ns*	*ns*
female	11.9 ± 1.5	12.0 ± 1.3	13.5 ± 1.3	*ns*	*0.02*	*0.04*
**Cerebellar GM**	101.7 ± 10.2	101.5 ± 13.8	105.1 ± 15.9	*ns*	*ns*	*ns*
male	107.6 ± 9.7	108.9 ± 8.7	105.3 ± 21.1	*ns*	*ns*	*ns*
female	97.7 ± 8.8	93.6 ± 14.3	104.9 ± 9.7	*ns*	*ns*	*0.05*
**Cerebellar WM**	24.0 ± 4.9	26.0 ± 6.6	31.7 ± 9.1	*ns*	*0.001*	*0.03*
male	25.40 ± 6.4	28.3 ± 5.9	32.7 ± 10.1	*ns*	*ns*	*ns*
female	23.0 ± 3.0	23.5 ± 7.0	30.7 ± 8.2	*ns*	*0.02*	*0.03*
**Hippocampus**	8.2 ± 0.8	8.5 ± 1.0	8.9 ± 0.8	*ns*	*0.009*	*ns*
male	8.5 ± 0.9	8.8 ± 0.9	9.1 ± 0.9	*ns*	*ns*	*ns*
female	8.0 ± 0.6	8.3 ± 1.1	8.8 ± 0.7	*ns*	*0.04*	*ns*

*ns, non-significant; GM, gray matter; WM, white matter.*

Group differences were also evaluated for regional brain volumes in relation to total intracranial volume. Relative cerebellar WM volume was lower in the PT-FGR and the PT-AGA groups as compared to the T-AGA group, *p* = 0.016 and 0.033, respectively. Remaining regional brain volumes in relation to total intracranial volume exhibited no differences between groups.

After adjustment for GA at birth, head circumference at term age correlated positively with global as well as with regional brain volumes within the respective PT groups. Fetal brain sparing was not associated with brain volumes. Weight deviation at birth was positively correlated to all regional brain volumes, but when GA at birth and sex were taken into account, only hippocampal volume remained significantly associated (*r* = 0.46, *p* = 0.05).

The presence of neonatal risk factors with a potential impact on brain growth, such as severe intraventricular hemorrhage grade III or periventricular hemorrhagic infarction, septicemia, and necrotizing enterocolitis, was not related to brain volumes. In univariate analysis, BPD was significantly associated with decreased volumes of GM, WM and cerebellar WM, but after adjustment for GA, birth weight deviation at birth and sex only GM volumes remained associated with BPD (*r* = 0.43, *p* = 0.01). Any stage of ROP was significantly associated with decreased volumes of WM, thalamus, and cerebellar GM, but after adjustment for GA, weight deviation at birth and sex, ROP remained significantly associated with cerebellar GM (*r* = 0.60, *p* = 0.02). Postnatal steroid treatment with betamethasone did not correlate with brain volumes.

Motor performance, cognitive outcome, behavior, and ADD were not associated with differences in any of the measured brain volumes. Sex had no influence on the relationship between regional brain volumes and neurodevelopmental outcome.

## Discussion

In this prospective study, we examined neurodevelopmental outcome and brain growth as determined by brain MRI at early school age in children born very preterm after early-onset FGR, and in matched preterm and term AGA controls. The PT-FGR group had a higher rate of cognitive impairment than PT-AGA subjects, however, we did not observe any corresponding differences in global or regional brain volumes as determined by MRI.

All children in the PT-FGR group had ARED blood flow in the umbilical artery prior to delivery and were actively delivered before 30 gestational weeks in order to prevent further worsening of fetal distress. We have previously reported that neonatal mortality, cerebral morbidity, and rate of cerebral palsy at 2 years of age in the PT-FGR group were comparable to those of children delivered very preterm due to other indications (Brodszki et al., 2009). However, subsequent follow-up showed that cognitive impairment at early school age was more prevalent in the PT-FGR as compared to the PT-AGA group, mainly due to decreased cognitive performance in growth restricted boys (Morsing et al., 2011).

Several studies have correlated adverse neurodevelopment with time of onset of growth restriction, (Baschat, 2014) severity of Doppler changes, (Schreuder et al., 2002) GA at delivery, (Baschat and Odibo, 2011), and neonatal morbidity (Yeh et al., 2004; Trittmann et al., 2013). The effect of fetal brain sparing was found to be associated with impaired cognitive outcome and to have negative effects on the brain (Scherjon et al., 2000). The majority of children in the present PT-FGR group had signs of fetal brain sparing, however, we did not find any relationship between redistribution of flow and brain volumes at early childhood.

The main result of the present study is the observation of smaller global and regional brain volumes in the preterm FGR group compared to the term AGA group, although no clear differences could be observed between the PT-FGR and PT-AGA groups. In previous studies, MRI performed at term-equivalent age (Tolsa et al., 2004) and at 12 months of age (Padilla et al., 2014) has shown reduced brain tissue volumes in infants born after FGR when compared to GA matched controls. It is important to note that subjects included in those studies had a considerably higher mean GA at birth, which, in absence of extreme prematurity, may increase the possibility of observing effects of FGR *per se*. The conflicting results might also reflect differences in pathophysiology between early-onset and late-onset FGR, and in the related clinical managements.

In our controlled study of preterm FGR subjects, hippocampal volume was positively correlated to weight deviation at birth. This is consistent with both experimental (Mallard et al., 2000) and clinical (Lodygensky et al., 2008) studies describing hippocampal vulnerability in growth restriction, possibly mediated by hypoxia and/or reduced supply of nutrients.

We observed sex-related differences in regional brain volumes in our study population. In comparison to the T-AGA reference group, female PT-FGR subjects had reduced thalamic, cerebellar, and hippocampal volumes, while male PT-FGR subjects had smaller WM volumes. These results are in accordance with literature that shows smaller WM volume at 8 years of age in preterm males (Reiss et al., 2004). Sex differences in brain morphology corresponding to those found in the present study were recently observed in a cohort of extremely preterm infants: female subjects had more abnormalities in the cerebellum and male subjects displayed delayed myelination (Skiold et al., 2014).

Among neonatal morbidities, BPD remained related to decreased GM volume after adjustment for GA and weight deviation at birth. The relationship between BPD and MRI abnormalities has been described previously. Brain abnormalities and delayed maturation in WM and thalamus were observed in MRI at term-equivalent age of PT infants with BPD. (Rose et al., 2014; Neubauer et al., 2015) In two other cohorts, BPD and exposure to postnatal dexamethasone resulted in smaller GM volume at term (Murphy et al., 2001) and in reduced total brain volume at adolescent age (Cheong et al., 2014). In our population, smaller GM volume was related to BPD but not to postnatal steroid treatment. The effect of postnatal treatment with betamethasone on brain growth has not been evaluated in PT infants as opposed to that of dexamethasone (Cheong et al., 2014). Speculatively, betamethasone may be less harmful to the brain tissue.

Further, any stage of ROP was related to reductions in cerebellar cortex volumes and these relationships remained significant after adjustment for confounders. In a recent study, presence of ROP was associated with smaller biometric measurements and higher prevalence of brain abnormalities on term-equivalent age MRI (Naud et al., 2017). We recently reported a relationship between any stage of ROP and reduced WM and cerebellar volume at term age as determined by MRI (Sveinsdottir et al., 2018). These findings suggest that common pathways may lead to impaired neural and neurovascular development in the brain and retina.

The main strengths of the present study are its prospective design and careful matching of study groups. The study has several limitations. Fetal Doppler measurements were not performed in the control groups. However, all subjects in the control groups were AGA at birth, which suggests that fetal blood flow impairment was less probable. Further, the study and control groups were relatively small and conclusions have to be drawn with caution. Thirty per cent of the children/parents did not consent to MRI examination which further reduced the strength of the study. However, cognitive and motor performance outcomes did not differ between children who declined and those who performed the MRI assessment in our study. The latter observation is reassuring in view of the finding in a recent meta-analysis, reporting that a greater loss to follow-up was correlated to higher rates of neurodevelopmental impairment (Guillen et al., 2012).

It is still unclear to which extent FGR with hemodynamic impairment prior to very preterm birth modifies or accentuates the risks of prematurity. Contrary to other studies, our data on the FGR group delivered very preterm did not show smaller regional brain volumes compared to those of matched PT-AGA controls. Cognitive impairment, more prevalent in the FGR group, was not related to brain volumes. One can speculate that the reduced brain volumes are not the most important pathophysiological factor connected to functional outcomes. Alternatively, reduced brain volumes seen at early ages, i.e., in neonates and in infancy, may normalize with age. We are currently analyzing data from diffusion tensor imaging (DTI), performed in parallel with the MRI volumetric study. Data on WM structure may provide important additional information about the effect of FGR on brain development.

## Ethics Statement

This study was approved by the Regional Research Ethics Committee at Lund University and examinations of the children were performed after their parents gave written informed consent.

## Author Contributions

EM conceptualized and designed the study, coordinated and supervised the data collection, carried out the statistical analysis, drafted the initial manuscript, and reviewed and revised the manuscript. KM, DL, and IB-B, conceptualized and designed the study, designed the data collection instruments, and critically reviewed and revised the manuscript. AK, MM, and JL, collected the data, carried out the initial analyses, and reviewed and revised the manuscript. All authors approved the final manuscript as submitted and agreed to be accountable for all aspects of the work.

## Conflict of Interest Statement

The authors declare that the research was conducted in the absence of any commercial or financial relationships that could be construed as a potential conflict of interest.

## Footnotes

1 http://surfer.nmr.mgh.harvard.edu

## Abbreviations

ADD: Attention deficit disorder
AGA: Appropriate for gestational age
ARED: Absent or reversed end-diastolic
BPD: Bronchopulmonary dysplasia
FGR: Fetal growth restriction
FIQ: Full scale IQ
GA: Gestational age
GM: Gray matter
MRI: Magnetic resonance imaging
PIQ: Performance IQ
PT: Preterm
ROP: Retinopathy of prematurity
ROI: Regions of interest
T: Term
VIQ: Verbal IQ
WM: White matter
